# Theoretical investigation of transgastric and intraductal approaches for ultrasound-based thermal therapy of the pancreas

**DOI:** 10.1186/s40349-017-0090-2

**Published:** 2017-05-03

**Authors:** Serena J. Scott, Matthew S. Adams, Vasant Salgaonkar, F. Graham Sommer, Chris J. Diederich

**Affiliations:** 1 Department of Radiation Oncology, Thermal Therapy Research Group, University of California, San Francisco, 1600 Divisadero Street, Suite H1031, San Francisco, CA 94143-1708 USA; 2 UC Berkeley – UC San Francisco Graduate Program in Bioengineering, California, USA; 3 Department of Radiology, Stanford University School of Medicine, Stanford, CA USA

**Keywords:** Ultrasound, Thermal ablation, Hyperthermia, Pancreas, Theoretical model

## Abstract

**Background:**

The goal of this study was to theoretically investigate the feasibility of intraductal and transgastric approaches to ultrasound-based thermal therapy of pancreatic tumors, and to evaluate possible treatment strategies.

**Methods:**

This study considered ultrasound applicators with 1.2 mm outer diameter tubular transducers, which are inserted into the tissue to be treated by an endoscopic approach, either via insertion through the gastric wall (transgastric) or within the pancreatic duct lumen (intraductal). 8 patient-specific, 3D, transient, biothermal and acoustic finite element models were generated to model hyperthermia (*n* = 2) and ablation (*n* = 6), using sectored (210°–270°, *n* = 4) and 360° (*n* = 4) transducers for treatment of 3.3–17.0 cm^3^ tumors in the head (*n* = 5), body (*n* = 2), and tail (*n* = 1) of the pancreas. A parametric study was performed to determine appropriate treatment parameters as a function of tissue attenuation, blood perfusion rates, and distance to sensitive anatomy.

**Results:**

Parametric studies indicated that pancreatic tumors up to 2.5 or 2.7 cm diameter can be ablated within 10 min with the transgastric and intraductal approaches, respectively. Patient-specific simulations demonstrated that 67.1–83.3% of the volumes of four sample 3.3–11.4 cm^3^ tumors could be ablated within 3–10 min using transgastric or intraductal approaches. 55.3–60.0% of the volume of a large 17.0 cm^3^ tumor could be ablated using multiple applicator positions within 20–30 min with either transgastric or intraductal approaches. 89.9–94.7% of the volume of two 4.4–11.4 cm^3^ tumors could be treated with intraductal hyperthermia. Sectored applicators are effective in directing acoustic output away from and preserving sensitive structures. When acoustic energy is directed towards sensitive structures, applicators should be placed at least 13.9–14.8 mm from major vessels like the aorta, 9.4–12.0 mm from other vessels, depending on the vessel size and flow rate, and 14 mm from the duodenum.

**Conclusions:**

This study demonstrated the feasibility of generating shaped or conformal ablative or hyperthermic temperature distributions within pancreatic tumors using transgastric or intraductal ultrasound.

## Background

Pancreatic cancer is a particularly severe disease, with a 5-year survival rate of about 6% in the United States [1]. It is the fourth most common cause of cancer-related deaths, causing 39,590 deaths per year in the United States [1]. Although surgery provides the best chance for cure [2, 3], most cases have progressed to advanced or locally advanced disease by the time of diagnosis [4, 5], and over 80% of patients are not candidates for resection [2, 5]. For patients who are not surgical candidates, prolongation of survival and palliative relief of symptoms are the major goals of medical treatment [3, 4, 6], with chemotherapy and radiotherapy as the most common interventions [2, 4]. Palliative care for advanced disease may include surgery, radiotherapy, biliary stenting, gastroduodenal stenting, analgesia, celiac plexus blockage, and prophylactic anticoagulants to care for conditions such as pain, jaundice, gastrointestinal obstruction, and venous embolism [2, 4, 6]. Thermal ablation has been shown to cause a reduction in tumor volume, to lower pain, and in some studies, to prolong survival [5, 7, 8].

Various ablative modalities have been considered for care of advanced pancreatic cancer, including RF ablation (RFA), microwave ablation, cryoablation, photodynamic therapy, high intensity focused ultrasound (HIFU), and irreversible electroporation, with HIFU and RFA receiving the most attention [5]. During thermal ablation, it is key to preserve sensitive tissues such as the duodenum and the peripancreatic vasculature, so a margin of tumor tissue is often left viable [7, 9]. In some studies, sensitive tissues were flushed with cold saline, and the vena cava could be covered with wet gauze during intraoperative interventions to prevent thermal injury [7, 9]. RFA of pancreatic cancer is usually performed during open surgery [9], but has also been performed using endoscopic approaches [10, 11]. The RF needle can be inserted through the working channel of an echoendoscope and into the pancreas through the wall of the stomach or duodenum under ultrasound guidance [10]. This endoscopic approach is less invasive than surgical and percutaneous approaches, and hence should result in fewer complications, but while the stomach and duodenum could potentially be water-cooled, this does not allow for active cooling of most sensitive tissues. Computed tomography (CT), magnetic resonance (MR), or ultrasound guidance and treatment monitoring may be applied to improve outcomes and reduce complications during thermal ablation [5, 8]. RF and other ablative therapies can be performed alongside the placement of biliary or duodenal stents as necessary to reduce complications and procedure time [5]. HIFU can be performed completely noninvasively with MR or ultrasound guidance, though limitations include bowel gas obstruction of the sonication path, which may preclude treatment of some targets, and the need to compensate for respiratory motion [8].

Endoscopic ultrasound applicators placed in the stomach or duodenum have previously been developed for thermal ablation of pancreatic tumors, applying either HIFU [12] or unfocused high intensity ultrasound ablation [13] techniques. Since transgastric and intraductal probes are advanced into the tumor itself, there are fewer concerns about breathing artifacts or motion of the applicator along the stomach wall than with other endoscopic ultrasound techniques. Transgastric and intraductal applicators can also access targets farther from the stomach wall than unfocused endoscopic probes placed within the stomach or duodenum. Catheter-based ultrasound ablation is also generally faster than HIFU, in which a small focal zone is scanned over the tumor, as the heated region covers a larger volume [14].

Small-diameter ultrasound applicators with tubular transducers could potentially allow for thermal therapy of pancreatic tumors with fewer limitations than HIFU or RF ablation. Such applicators have been applied in the past in the prostate, liver, bone, heart, and brain using catheter or balloon-based cooling, and are in clinical use for hyperthermia [14–21]. These flexible applicators are advanced directly into or adjacent to the tissue to be treated, and achieve 15–21 mm of thermal penetration within 5–10 min with spatial control along the length and angle of the applicator [15, 22–24]. For applications in the pancreas, the ultrasound applicator could be advanced through the working channel of an echoendoscope, then through the stomach wall, duodenal wall, pancreatic duct, or biliary duct, and directly into the tumor under ultrasound imaging guidance. Intraductal ablation can be done using techniques similar to those applied in prior treatments of the pancreas and biliary ducts using RFA [25–27] or planar ultrasound applicators [28]. Transgastric ablation could be performed using techniques similar to those of needle-based RF ablation [29, 30] and catheter-based cryotherm ablation, which uses a 1.8 mm probe [10, 31]. Sectored ultrasound transducers can be used for sparing of adjacent sensitive tissues, by providing directionality that RF, microwave, and laser ablation lack.

The goal of this study is to apply theoretical models to evaluate the feasibility of ultrasound-based thermal therapy using endoscopic approaches to deliver interstitial or catheter-based ultrasound devices specific for this application. Theoretical modeling techniques that have been previously tested and validated broadly in both bone and soft tissue [16, 17, 32, 33] are applied in this theoretical study to perform a preliminary investigation. Numerical models of several patient cases are created to assess various approaches, applicator configurations, and treatment parameters. Both ablation and hyperthermia, which has been shown to improve the effects of chemotherapy and radiation in the treatment of pancreatic cancer [34, 35], are considered. Parametric studies are performed to determine the necessary safety margins for preservation of major blood vessels and the duodenum, and to investigate the effects of the acoustic absorption coefficient and blood perfusion rates on treatment outcomes.

## Methods

### Endoscopic ultrasound devices

The ultrasound applicators considered in this study are to be deployed through the working channels of endoscopic probes, which are often up to 3.2 mm inner diameter (ID) [36]. Two different approaches are applied: direct insertion of an ultrasound applicator into the tumor through the wall of the stomach or duodenum, and insertion of a more flexible applicator through the pancreatic duct. This can be performed using routes commonly employed during endoscopic ultrasound-guided biopsy and pancreatic duct stenting, and techniques previously employed for transgastric ablation of the pancreas [10, 29–31]. Both hyperthermia and ablation are considered for the intraductal approach. Only ablation is considered for the transgastric approach, to balance the risks associated this slightly more invasive technique with a more thorough and direct treatment.

Both types of applicators consist of 2–3 tubular ultrasound transducers (7 MHz; 1.2 mm outer diameter (OD); 7.5, 10, or 15 mm long) mounted on the distal tip of an applicator. This device configuration is modeled after those commonly used in similar interstitial ultrasound applicators [14, 33, 37], with a slightly smaller diameter to minimize damage to the stomach wall and for easier maneuverability within the pancreatic duct. These transducers, which radiate radially outward, can be sectored to attain directional control of the acoustic output, such that the transducers radiate only to one side [37]. 210°, 270°, and 360° active sectors were considered in this study. The applicators are water-cooled for acoustic coupling and to prevent thermal damage to the transducers.

**Fig. 1:**
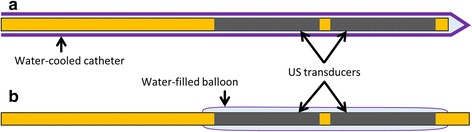
Diagram of transgastric (**a**) and intraductal (**b**) applicators for ultrasound-based thermal therapy of the pancreas. Transgastric applicators are operated from within a water-cooled catheter (1.6 mm ID, 2.11 mm OD), while intraductal applicators have a thin water-cooled balloon (2.2 mm OD) around the transducers. 2–3 transducers can be mounted on each applicator, with an outer diameter of 1.2 mm and a length of 7.5, 10, or 15 mm

The transgastric applicators are inserted into the tumor through the stomach or duodenal wall, and are deployed within a water-cooled catheter (Fig. 1a), similar to catheter-based ultrasound applicators designed for percutaneous insertion [37, 38]. The plastic catheter (1.6 mm ID, 2.11 mm OD) around the transgastric applicator is modeled after the cooling flow lumen and wall thicknesses of catheters for interstitial applicators [39, 40], and is assumed to have an ultrasound attenuation coefficient of 43.9 Np/m/MHz [39]. Intraductal applicators would be advanced through the biliary or pancreatic duct in order to access the tumor. The transducers are surrounded by a single distensible water-cooled balloon (2.2 mm OD, 12.7 μm thick, Fig. 1b), similar to that used in transurethral prostate ablation [41]. The applicator is designed to be small enough to fit within the duct [42] while maintaining a layer of cooling water around the transducers. It is similar in size to pancreatic stents [43] and, when uninflated, intraductal ultrasound imaging probes [44]. The balloon around the intraductal applicator is assumed to be so thin as to cause negligible acoustic attenuation.

### Acoustic and biothermal simulations

Heat transfer through physiological tissues was modeled using Pennes bioheat equation [45]:

1 $$ \rho c\frac{dT}{dt} = \nabla \cdot \mathrm{k}\nabla \mathrm{T} - {\upomega \mathrm{c}}_b\left( T-{T}_b\right)+ Q $$

where *ρ* is density (kg/m^3^), *c* is specific heat (J/kg/°C), *T* is temperature (°C), *t* is time (s), k is thermal conductivity (W/m/°C), ω is the blood perfusion rate (kg/m^3^/s), *Q* is the heat deposition due to ultrasound (W/m^3^), the subscript *b* refers to blood, and capillary blood temperature *T*_*b*_ is assumed to be 37 °C. To approximate the effects of heating-induced microvascular stasis, which occurs at a thermal dose of around 300 EM43°C [16], blood perfusion rates in all tissues were assumed to reduce to zero at a temperature of 54 °C, which was found in this computational study to correspond to 300 EM43°C at the acoustic intensities and durations typically applied. The material properties of the various tissues considered are specified in Table 1.

**Table 1:** Material properties of tissues. ƒ represents frequency, in units of MHz

Medium	Density (kg/m^3^)	Attenuation (Np/m)	Thermal conductivity (W/m/K)	Specific heat (J/kg/K)	Perfusion rate (kg/m^3^/s)
Tumor	1050^a^	10 ƒ [52, 65]	.51^a^	3200^a^	4.5 [51, 66–68]
Pancreas	1050 [52]	11.9 ƒ^0.78^ [52]	.51 [52]	3200 [69]	10 [52]
Duodenal wall	1050 [52]	5 ƒ	.53 [52, 69]	3700 [69–71]	16.7 [52, 69]
Stomach wall	1060 [52, 69]	5 ƒ	.54 [52, 69]	3700 [69]	6.8 [52, 69]
Water, pancreatic juices, and gastric contents	1010 [52, 72]	0.025 ƒ^2^ [52]	.58	4000	0
Blood	N/A	1.6 ƒ^1.21^ [52]	N/A	3600 [52]	N/A
Liver	1050 [52]	4.5 ƒ [16]	.51 [52]	3600 [16]	15 [16]
Kidney	1050 [52]	6 ƒ [52]	.54 [52]	3700 [52]	68.9 [52]
Spleen	1050 [52]	13.2 ƒ [52]	.54 [52]	3700 [52]	22.1 [52]
Bone	1420 [52]	105 ƒ [52]	.38 [52]	1700 [73]	0.89 [74]
Renal calcifications	1990 [52]^b^	105 ƒ [52]^b^	.5 [52]^c^	1300 [52]^b^	0
Bowel	1040 [52]	5.6 ƒ [52]	0.56 [52]	3600 [69]	16.4 [52]
Other soft tissue	1050 [52]	6.4 ƒ [52]	.49 [52]	3400 [52]	3 [52]

Properties of normal pancreatic tissue were used for some pancreatic tumor properties (^a^). The acoustic attenuation of various neoplasms was used to estimate the attenuation in pancreatic tumors. Because the specific heat of water is very different from those of tissue and biological fluids, an intermediate value was used for the specific heat of water, pancreatic juices, and gastric contents. Attenuation of the stomach and duodenal walls were estimated from measurements performed in porcine stomach tissue. Properties for whole or cortical bone (^b^) were used for some calcification properties, and properties for calcified arterial plaques were used for another (^c^)

Tissues that reached 52 °C were considered to be ablated [46], and hyperthermia simulations were performed with an aim of maintaining temperatures of 40–47 °C throughout the target volume. Sensitive tissues, including the stomach wall, intestines, blood vessels, liver, kidneys, spleen, and bones, were to be kept below a safety threshold of 45 °C [47].

The acoustic heat deposition from a tubular ultrasound source can be modeled as a radially radiating intensity profile well-collimated to the length and sector angle of the transducer [33, 41]:

2 $$ Q=2\alpha {I}_s\frac{r_t}{r}{e}^{-2{\displaystyle {\int}_{r_t}^r\mu d{r}^{{\textstyle \hbox{'}}}}} $$

where *α* is the ultrasound absorption coefficient (Np/m), *I*_*s*_ is the acoustic intensity on the transducer surface (W/m^2^), *r*_*t*_ is the transducer radius (m), *r* is the radial distance from the central axis of the transducer (m), and *μ* is the ultrasound attenuation coefficient (Np/m). The ultrasound absorption coefficient is assumed to be equivalent to the ultrasound attenuation coefficient, with scattered energy locally absorbed.

Heat transfer in tissue was modeled using COMSOL Multiphysics 4.4 (COMSOL, Inc., Burlington, MA) in conjunction with MATLAB (Mathworks, Inc., Natick, MA). An initial tissue temperature of 37 °C was assumed for all simulations. A Dirichlet boundary condition constrained the outermost boundaries of the tissue volume, far from the heated region, to 37 °C. Blood flow through larger vessels [48], as well as water cooling of the catheter and balloon, are modeled using convective boundary conditions:

3 $$ -\widehat{n}\cdot \left(- k\nabla \mathrm{T}\right)= h\left({T}_f- T\right) $$

where $\widehat{n}$ is the unit vector normal to the vessel, catheter, or balloon surface, *h* is the heat transfer coefficient (W/m^2^/°C), and *T*_*f*_ is the fluid temperature, as specified in

Table 2. To simplify the modeling of the vessels in the patient-specific study, which had non-constant diameters and complex geometries, the vessels were assigned a heat transfer coefficient based on size and flow rate. Unique heat transfer coefficients were calculated for the aorta and vena cava. In the remaining vasculature the heat transfer coefficient was calculated to be approximately 750 W/m^2^/°C for large vessels such as the portal vein, superior mesenteric vein, etc., and 1000 W/m^2^/°C for smaller vessels such as the splenic artery, superior mesenteric artery, hepatic artery, etc.

**Table 2:** Heat transfer coefficients and fluid temperatures for convective flow boundaries

Surface	*h* (W/m^2^/°C)	*T*_*f*_ (°C)
Transgastric catheter	1000 [16, 75]	22
Intraductal balloon	500 [13, 63]	22
Aorta	511	37
Vena cava	184	37
Large vessels	750	37
Small vessels	1000	37

Heat transfer coefficients for blood vessels are calculated based upon vessel sizes, which were measured in CT scans, and flow rates [76–82] as described by Haemmerich et al. [48]. “Large vessels” included the portal vein, superior mesenteric vein, etc. “Small vessels” included the superior mesenteric artery, splenic artery, hepatic artery, etc

Convergence tests were performed to select mesh sizes and time steps small enough for accuracy. The finite element mesh size was limited to a maximum of 0.45 mm on the applicator, and no more than 7–7.5 mm overall, with a finer mesh on highly heated volumes, a wider mesh on the outer tissue boundaries, and gradual transitions in mesh size over space. An implicit transient solver with variable time stepping was used in ablation simulations as necessary in order to track the dynamic spreading of high temperatures over short treatment intervals. Initial time steps were small (<1 s), and gradually increased automatically to no more than 15 s as the solution converged. When determining the amount of time it takes for sensitive tissue to heat to dangerous temperatures in parametric studies, maximum time steps were limited to 2–15 s at time points when the tissue was expected to approach the temperature threshold. Hyperthermia distributions were calculated using a steady-state solver under steady-state conditions, which assume that target temperatures are achieved within a short interval and maintained for a typical 30–60 min treatment session.

### Patient-specific models

**Fig. 2:**
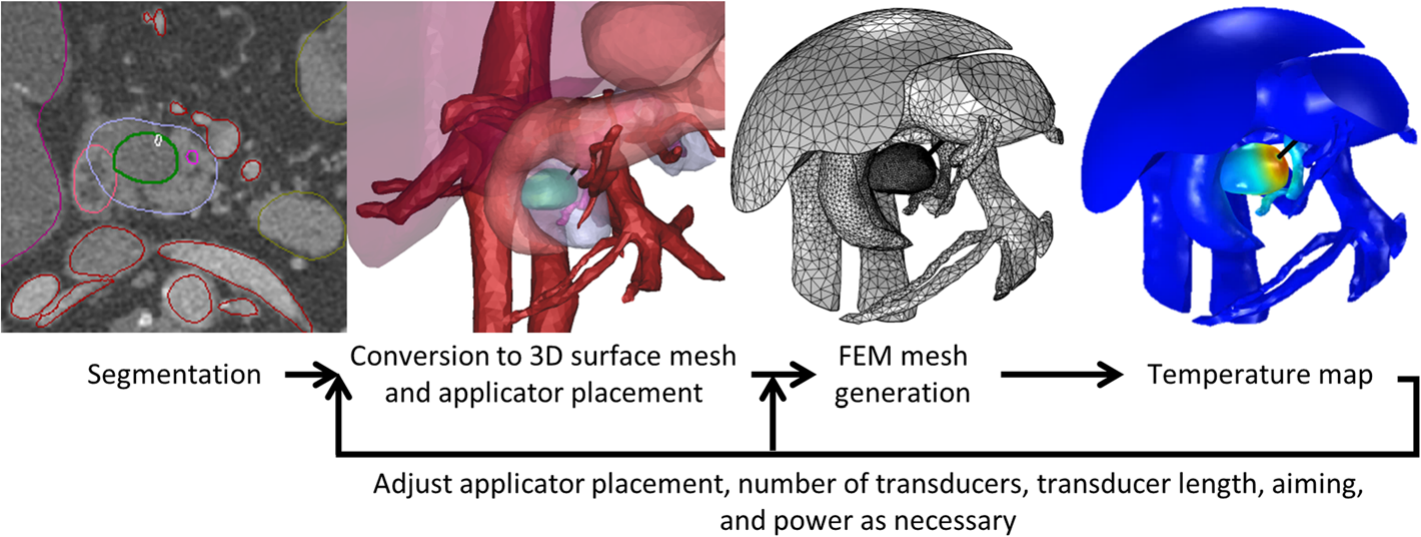
Process used for creation of 3D patient-specific models

To investigate approaches for transgastric and intraductal thermal therapy using tubular ultrasound transducers, eight 3D patient-specific models were developed. The 3D models were made by segmenting CT scans and then creating 3D finite element meshes based on individual patient anatomies (Fig. 2). Individual organs and structures, including tumors and blood vessels, were identified. Applicator positions and treatment parameters were selected using an empirical, iterative approach to maximize tumor coverage without damaging sensitive anatomy. Finite element modeling was performed to calculate temperatures throughout the tissue volume. Ablation was modeled using time-dependent simulations, with a proportional integral (PI) controller to determine applied powers, while hyperthermia was modeled at steady state, with constant powers selected empirically.

To create the models, CT scans of six patients with tumors (1.9–4.8 mm long along the applicator axis, 3.3–17.0 cm^3^, Table 3) in the head (*n* = 4), body (*n* = 1), or tail (*n* = 1) of the pancreas were segmented using a combination of manual and semi-automatic techniques. Note the UCSF Internal Review Board (IRB) considers the use of de-identified data (images used herein) without a key back to the subject as “Not Human Subjects Research”, and does not require approval. All organs near each target were selected for segmentation, and could include the tumor, pancreas, pancreatic duct (if visibly distended), duodenal wall, stomach wall, gastrointestinal contents, liver, kidney, spleen, vertebrae, and bowel. Thermally significant blood vessels, such as the aorta, vena cava, portal vein, superior mesenteric vein, splenic vein, renal veins, and superior mesenteric artery, were also segmented. Image segmentation and finite element mesh generation were performed using the Mimics Innovation Suite (Materialise NV, Leuven, Belgium), as illustrated in Fig. 2. The finite element mesh was imported into COMSOL Multiphysics, where acoustic and thermal modeling of ablation and/or hyperthermia was performed.

**Table 3:** Treatment parameters for patient-specific cases

Case	Tumor location	Tumor length (cm)	Tumor volume (cm^3^)	Approach	Treatment	Applicator configuration	Treatment time (min)	Mean power (W)	% volume treated
1	Body	4.8	17.0	Transgastric	Ablation	2x10 mm, 270°	20	10.1–11.9	60.0
2	Body	4.8	17.0	Intraductal	Ablation	2x10 mm, 210°	30	8.8–11.6	55.3
3	Tail	1.9	3.3	Transgastric	Ablation	2x7.5 mm, 270°	10	13.3	72.7
4	Head 1	2.5	4.4	Intraductal	Hyperthermia	2x15 mm, 360°	N/A	3.8	89.9
5	Head 1	2.5	4.4	Intraductal	Ablation	2x15 mm, 360°	8	9.7	83.3
6	Head 2	2.0	4.4	Transgastric	Ablation	2x10 mm, 360°	9	10.5	81.6
7	Head 3	3.9	11.4	Intraductal	Hyperthermia	3x15 mm, 360°	N/A	3.5	94.7
8	Head 4	3.2	7.9	Transgastric	Ablation	2x15 mm, 270°	10	10.0	67.1

Four different tumors in the head of the pancreas were modeled, and are numbered. Tumor length is defined as the tumor dimension along the applicator axis. The applicator configuration gives the number of transducers, the length of the transducers, and the sector size. Regions over 52 °C for ablation and 40 °C for hyperthermia are considered to be treated. Powers for Cases 1 and 2 were adjusted at each applicator position. Power for each time-dependent ablation study was varied continuously by a PI controller, so mean power is reported. Hyperthermia treatments were modeled as steady-state simulations, so a single value for power was simulated, and a specific treatment time is not applicable

An iterative approach was employed for each patient case to determine appropriate treatment parameters, such as applicator position, transducer length, transducer sector angle, power, and treatment time. Power levels for hyperthermia were empirically selected to raise a maximum volume of the tumor to 40–47 °C while maintaining critical anatomy, such as the duodenum and blood vessels, under 45 °C. In transient ablation simulations, a PI controller (k_p_ = 0.375 W/°C, k_i_ = 0.003 W/°C/s) was used to maintain maximum temperatures in the tumor volume of 80 or 85 °C, with a lower target temperature used in cases with longer treatment durations and in cases with sensitive tissues in proximity to the most heated regions. For clinical implementation of such a controller, temperature measurements from MR temperature imaging or needle-based temperature probes could be employed. Ablation treatments, which were simulated using a time-dependent model, were considered complete, and treatment was ended, when the full tumor volume reached a lethal temperature of 52 °C, or when sensitive anatomy to be preserved neared 45 °C, whichever came first.

Transgastric approaches were modeled in cases with an available insertion path from the stomach or duodenum to the tumor that did not transverse any sensitive anatomy. Intraductal approaches were modeled in cases in which most of the tumor volume was within 1.5 cm of the pancreatic or bile duct. In cases in which the tumor is on one side of the bile duct, a transgastric applicator can be placed through the center of the tumor, and an intraductal applicator can use sectored transducers to heat to one side. However, the far side of a tumor adjacent to the duct may be too far from an intraductal applicator for it to be treated effectively, so for this reason, the tumors considered in Cases 6 and 8 were modeled only with a transgastric approach. To simulate realistic applicator positions as delivered through the working channel of an endoscope, the transgastric applicators were inserted through the stomach or duodenal wall at an acute angle to the tangent plane of the wall. In Cases 1, 2, and 8, in which the stomach or duodenum was close to the targeted volume and in danger of thermal damage, the stomach and duodenum were assumed to be filled with 22 °C cooling water 2–5 min before treatment began. This cooling was modeled using an initial temperature condition within the stomach and duodenum of 22 °C, and an initial temperature of 37 °C elsewhere.

Hyperthermia was considered only for tumors near the duodenum that could be treated intraductally using a single applicator position. To warrant the invasiveness of a transgastric approach, only the aggressive treatment of ablation was considered for such cases. The tail of the pancreas was judged to be too far from the duodenum to be accessed endoscopically through the pancreatic duct. To be treated with hyperthermia, which lasts on the order of an hour, the tumor also had to be small enough to treat the full volume using a single applicator position, as multiple positions would require excessive amounts of time.

### Parametric studies

Parametric studies were performed to investigate necessary treatment parameters as a function of distance from critical blood vessels, distance from the duodenum, the acoustic absorption coefficient, and blood perfusion rates. Simple geometric models and meshes were created in COMSOL to represent the various tissues, with the same meshing and modeling parameters as the patient-specific models. The applicator was positioned in the center of a large cylinder of soft tissue. A PI controller (k_p_ = 0.375 W/°C, k_i_ = 0.003 W/°C/s) was used to maintain maximum tissue temperatures of 80 °C.

### Parametric studies of preservation of blood vessels

A wide variety of critical blood vessels are in close proximity to the pancreas, including but not limited to the aorta, the vena cava, the portal vein, the superior mesenteric vein and artery, the splenic vein and artery, the renal veins and arteries, the common hepatic artery, the right gastroepiploic vein, the celiac artery, and the left gastric artery. A parametric study was performed to evaluate the effect of any errors in estimation of the heat transfer coefficients of the vessels and to determine the distance between the applicator and the vessels necessary for the vessels to be fully preserved (*T* < 45 °C).

**Fig. 3:**
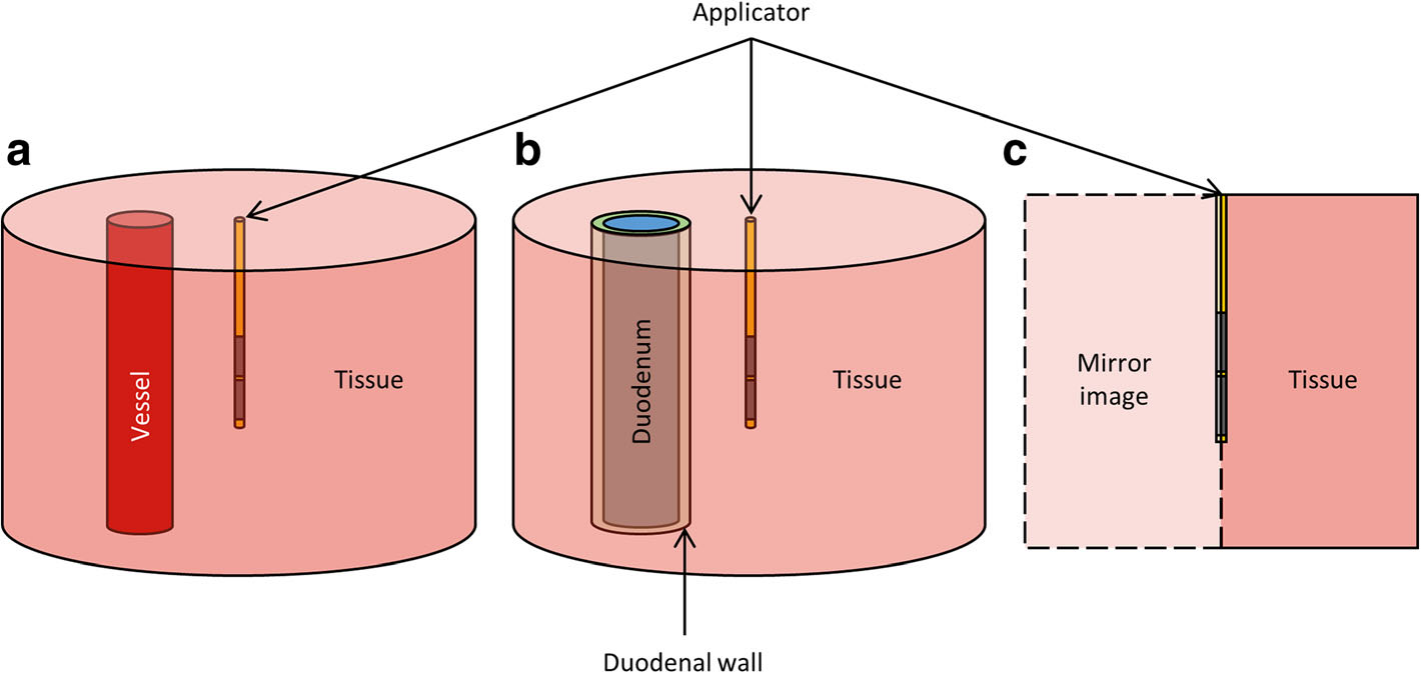
Diagram of geometries of parametric studies, showing the applicator in the center of a cylinder of tissue. Geometries for studies of blood vessel and duodenal heating include two concentric cylinders representing the inner and outer surfaces of a blood vessel (**a**) or duodenal wall (**b**). In 2D studies of the impacts of attenuation and perfusion, the temperature distributions in the axisymetric geometry was rotated about the central axis to represent a 3D cylinder (**c**)

In these 3D models, the blood vessels were represented by an 8 cm high tube oriented parallel to the applicator (Fig. 3a). The transgastric applicator considered had two 1 cm long, 360° transducers, which were positioned in the center of a 12 cm OD, 8 cm high cylinder of tissue surrounding both the applicator and the vessel (Fig. 3a). The aorta and vena cava were represented by 18 mm ID, 22 mm OD hollow cylinders; large vessels such as the portal vein by 11 mm ID, 13 mm OD hollow cylinders, and small vessels such as the superior mesenteric artery by 5 mm ID, 5.8 mm OD hollow cylinders. The distances between the vessels and the applicators reported in this study are measured from the outer surface of the vessel to the center of the applicator.

A wide variety of heat transfer coefficients, ranging from 46 or 184 kg/m^3^/s up to 1500 or 3000 kg/m^3^/s, were applied for each vessel size bracket in this parametric study. The range considered is far wider than the heat transfer coefficients calculated for the vessels in each size grouping. Such a wide range is modeled so that the effects of vessel size on temperature can be observed as a function of the heat transfer coefficient.

### Parametric study of duodenal heating

Because the pancreas is in such close proximity to the stomach and duodenum, a parametric study was performed to assess techniques for preserving these tissues. As most tissue properties are similar for these two materials, and the majority of pancreatic tumors arise in the head of the pancreas [6] which is near the duodenum, heating of the duodenum was modeled in this study. The effects of the distance from the applicator to the duodenum, the use of sectored transducers, and water-cooling of the duodenum on the peak temperatures generated in the duodenum were evaluated. 360° and 270° sector angles were considered, with the 270° sectors directed away from the duodenum. The applicator was placed 2–30 mm from the duodenum, as measured from the center of the applicator to the outer surface of the duodenal wall. To water-cool the duodenum, it was assumed that the duodenal lumen was filled with 22 °C water immediately before heating began, and that the water was left in the duodenum and gradually warmed by the surrounding tissue. In cases without cooling, the initial temperature of the duodenal lumen was assumed to be 37 °C. In cases with cooling, the duodenal lumen was assumed to have an initial temperature of 22 °C, and all surrounding tissues were assumed to have an initial temperature of 37 °C.

In these 3D models, a transgastric applicator with two 1 cm long transducers was positioned in the center of a large cylinder of tissue (8 cm high, 12 cm diameter). Two concentric 8 cm high cylinders parallel to the applicator represented inner and outer surfaces of the duodenum, which was assumed to have an outer diameter of 25 mm (Fig. 3b) [49]. Duodenal wall thickness has been reported as 1.5–3 mm [49, 50], so a wall thickness of 3 mm was assumed to avoid underestimation of duodenal heating. The contents of the duodenum were modeled as water.

### Parametric study of absorption and perfusion

As the acoustic properties and blood perfusion rates of pancreatic tumors have not been widely reported in the literature, a parametric study was performed to evaluate the effects of ranges of acoustic attenuation and blood perfusion rates on ablation outcomes. The maximum lesion diameter (T > 52 °C) was calculated for a variety of tissue attenuations (35–85 Np/m at 7 MHz) and perfusion rates (0–10 kg/m^3^/s), ranges chosen to encompass a variety of recorded tumor perfusion rates [51], necrotic tumor cores, and a variety of attenuation values common in healthy and cancerous soft tissues [52]. All other material properties for pancreatic tumors were set to those of pancreatic tissue, as shown in Table 1. An applicator with two 15 mm long 360° transducers was considered. It was placed with the transducers in the center of a 9 cm high, 5 cm radius cylinder of tissue (Fig. 3c), and heating was performed for 10 min, considering both transgastric and intraductal applicators.

## Results

### Patient-specific models

Hyperthermia and/or ablation treatments of 6 pancreatic tumors were simulated using intraductal and/or transgastric approaches. Four tumors were in the head of the pancreas, one was in the body, and one was in the tail. Eight treatments were simulated: four intraductal and four transgastric approaches, with six ablations and two hyperthermia treatments. The tumor sizes, treatment parameters, and treatment outcomes for the 8 cases are summarized in Table 3. 67.1–83.3% of the volumes of four small and medium-sized tumors (3.3–7.9 cm^3^) could be ablated within 10 min using one applicator position. In contrast, only 55.3 or 60% of the volume of a larger (17.0 cm^3^) tumor that required multiple repositionings of the applicator could be ablated within 20 min using intraductal or transgastric approaches, respectively. Intraductal hyperthermia was able to treat relatively large portions (89.9–94.7%) of two small and medium sized tumors (4.4 & 11.4 cm^3^). In all cases, sensitive anatomy was kept at temperatures of no more than 45 °C (Table 4).

**Table 4:** Maximum temperature (°C) in tumors and the sensitive anatomy modeled in the eight cases considered

Case	Treatment	Tumor	Duodenum	Stomach	Blood vessels	Liver	Kidney	Bone	Spleen	Intestine
1	Ablation	83.6	N/A	44.6	44.7	N/A	N/A	N/A	N/A	N/A
2	Ablation	84.4	N/A	43.2	45.0	N/A	N/A	N/A	N/A	N/A
3	Ablation	84.5	N/A	40.5	44.3	N/A	43.3	N/A	40.5	N/A
4	Hyperthermia	47.1	38.0	37.6	39.0	37.1	N/A	37.4	N/A	N/A
5	Ablation	80.1	40.0	38.3	44.6	37.2	N/A	38.0	N/A	N/A
6	Ablation	79.8	44.7	37.4	39.2	37.4	N/A	N/A	N/A	38.1
7	Hyperthermia	47.1	42.9	37.1	39.7	37.8	37.3	38.7	N/A	N/A
8	Ablation	80.1	42.6	37.1	42.5	N/A	37.0	38.1	N/A	N/A

**Fig. 4:**
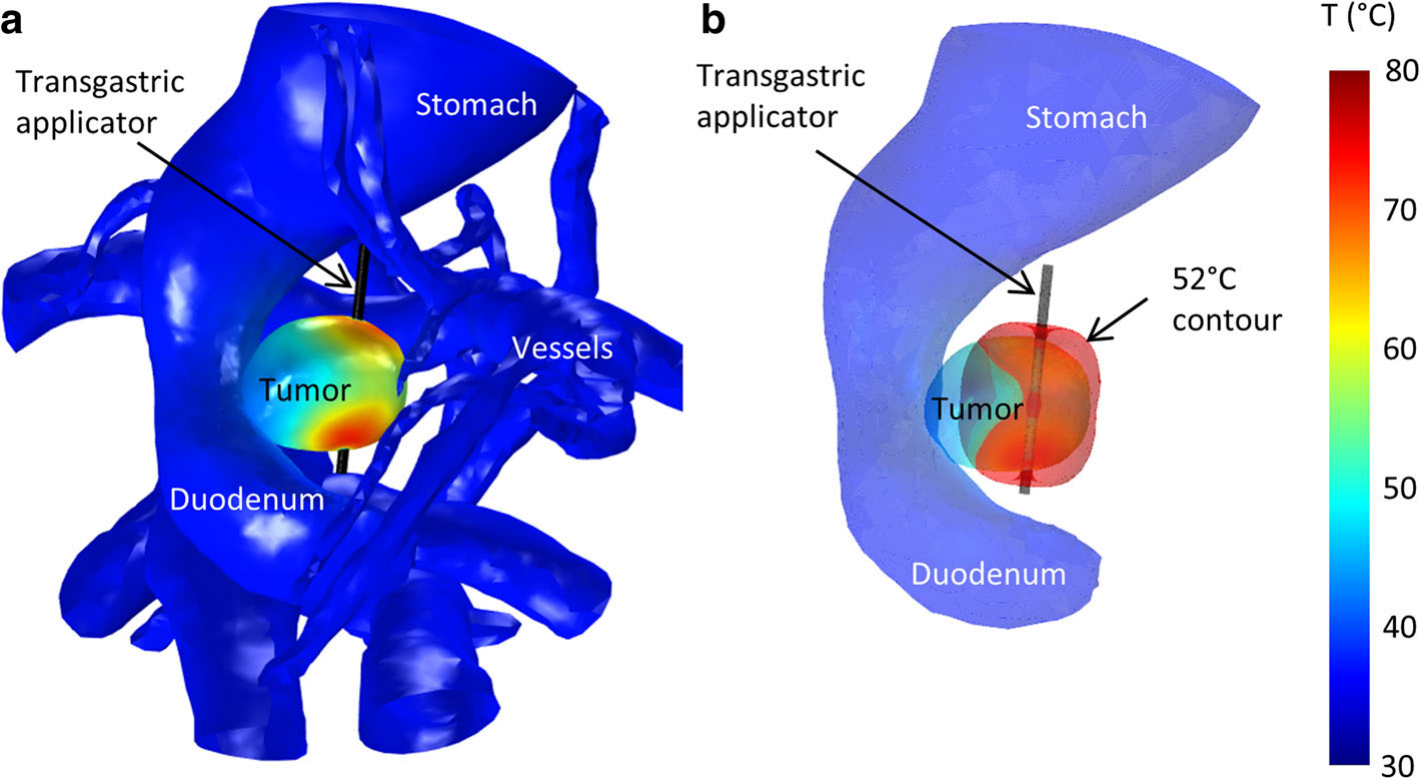
3D images showing placement of applicator off-center within tumor to avoid heating of the duodenum (Case 6). **a** Map of temperatures on the tumor, duodenum, stomach, and blood vessels. **b** 52 °C contour shown relative to the positions of the tumor, duodenum, and stomach

Both 360° and sectored transducers were used. To protect sensitive anatomy while heating in all directions in order to treat larger volumes, 360° applicators were placed off-center within the tumors, away from sensitive tissues (Fig. 4), in Cases 4–6. In four cases, transducers with wide sector angles of 210 or 270 °C carefully directed away from sensitive anatomy were utilized.

**Fig. 5:**
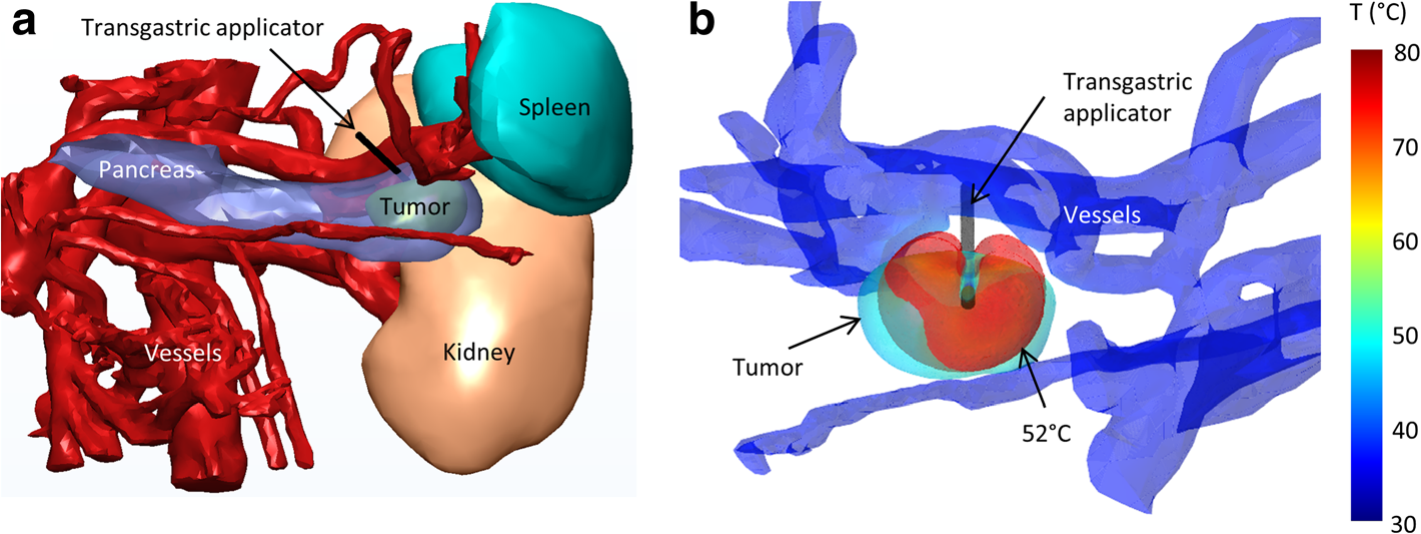
**a** 3D rendering of the anatomy in Case 3, showing the position of the vessels, kidney, and spleen around the tumor. **b** Map of temperature on tumor and blood vessel surfaces, with the 52 °C contour (red) and the 270 °C applicator shown

In Case 3, the pancreatic tail tumor was surrounded by several critical organs, including the right kidney, the spleen, and the splenic vein. To protect the blood vessels, a 270° directional applicator was placed adjacent to the vessels closest to the tumor, and the acoustic energy was directed away from these vessels (Fig. 5). A small portion of the tumor in the direction of the non-active sector was heated through conduction, but there was little heating penetration in the non-active direction, and minimal heating in the direction of the nearby vessels. To avoid renal heating, the applicator was placed perpendicular to the kidney surface, though not deep enough to puncture it, so that the well-collimated acoustic beam would not enter the kidney.

**Fig. 6:**
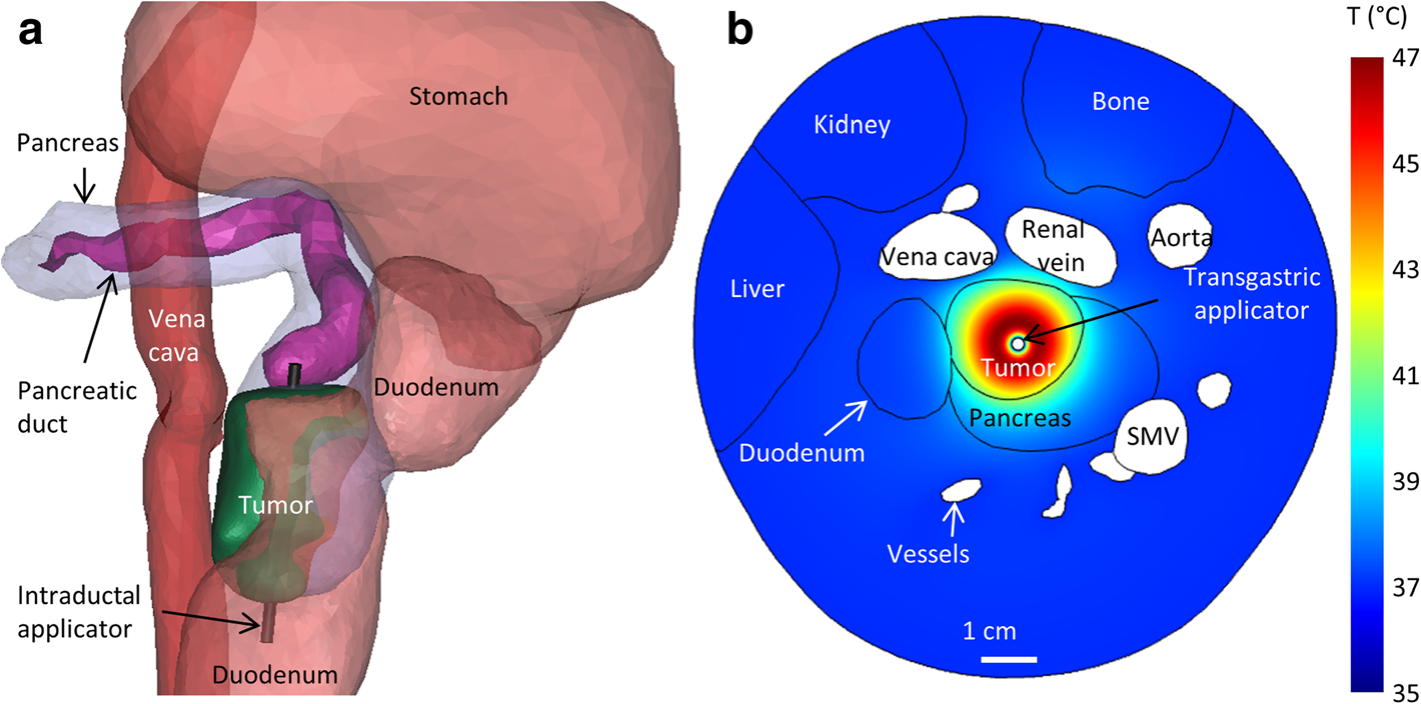
**a** 3D image of anatomy showing position of tumor between vena cava and duodenum (Case 7), with several organs not shown for clarity. **b** 2D map of temperatures during intraductal hyperthermia treatment. Superior mesenteric artery is abbreviated SMV

In Case 7 the duodenum, renal vein, and vena cava were located in close proximity to a tumor in the head of the pancreas, with the duodenum on the right side of the tumor and these two vessels immediately posterior to the tumor (Fig. 6). As directional heating could not be applied to direct all heat away from both of these structures without leaving significant portions of the tumor untreated, this case was modeled using only hyperthermia and a 360° applicator. 97.3% of the tumor volume was heated to a target temperature of 40 °C or higher, with a maximum tumor temperature of 47.1 °C near the applicator. The maximum temperatures on nearby sensitive anatomy were 42.9, 39.7, 38.7, 37.8, and 37.3 °C in the duodenum, blood vessels, bone, liver, and kidney, respectively.

**Fig. 7:**
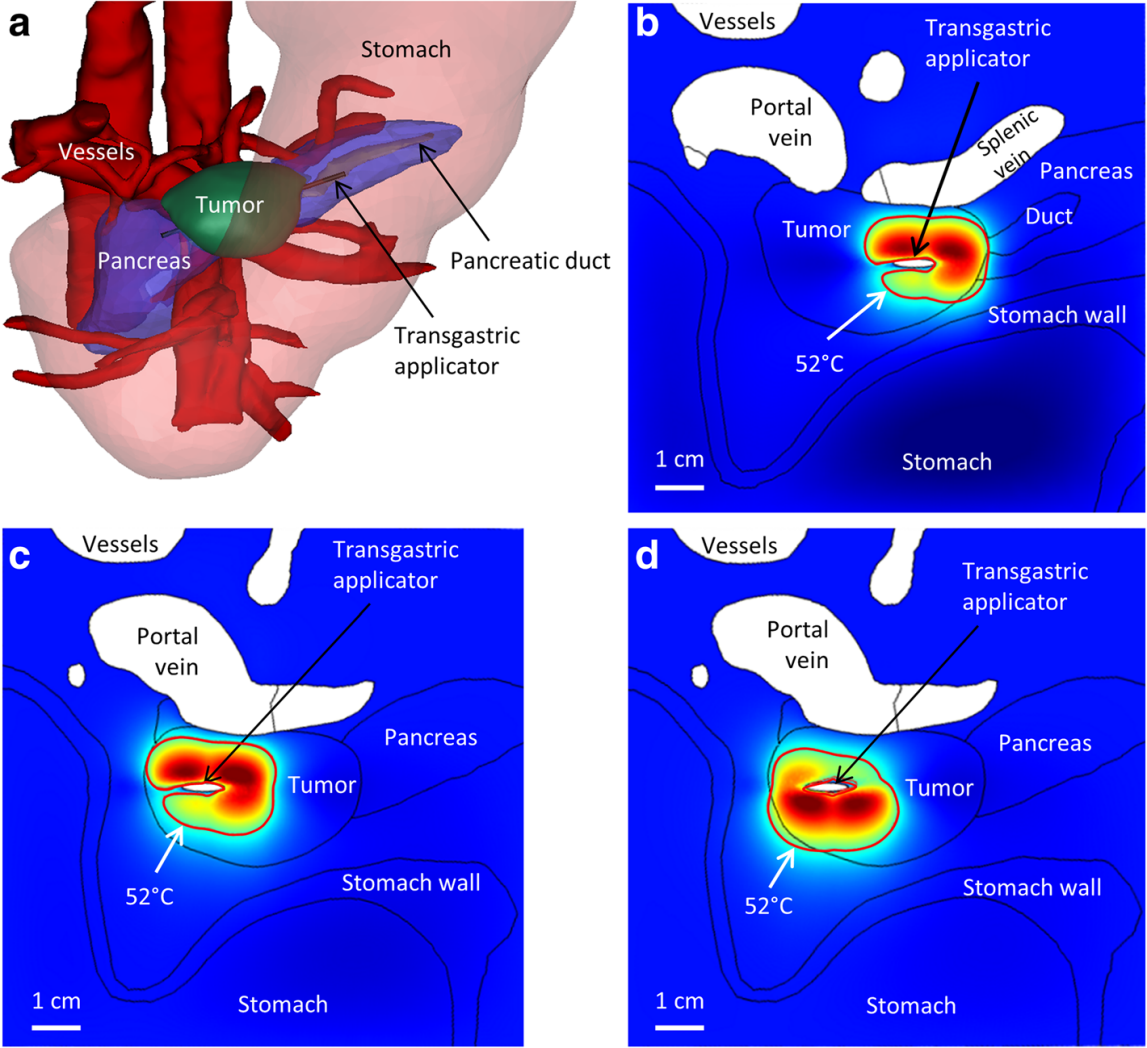
**a** 3D image of anatomy showing position of large tumor (Cases 1&2) between the stomach wall and several major blood vessels (white). **b**-**d** Axial 2D maps of temperatures at the end of ablations at each of three position of the transgastric applicator (Case 1)

Both transgastric and intraductal approaches were considered to treat a large (4.8 cm long) tumor in the head of the pancreas. As the stomach and major vessels were very close to the tumor, 270° sectored applicators were employed to minimize energy directed towards these sensitive tissues. To cover this tumor, the applicator, which contained two 10 mm transducers, was inserted to two depths for both approaches, and rotated to 1–2 positions at each depth. There were a total of 3–4 applicator positions and rotations over the course of each of the transgastric and intraductal ablations. To provide additional cooling for both approaches, the stomach was assumed to have been filled with 22 °C water before the start of the first ablations, in which the applicator was placed closest to the stomach wall. Hyperthermia was not performed in this case because treatment at 4 discrete positions for 30–60 min each would take an unreasonable amount of time. The transgastric approach, employing 3 applicator positions, allowed for 10.2 cm^3^ of the 17.0 cm^3^ tumor to be ablated over 20 min, with maximum stomach and blood vessel temperatures of 44.7 and 44.6 °C, respectively (Fig. 7). The intraductal approach, employing 4 discrete applicator positions, allowed for 9.4 cm^3^ of the 17.0 cm^3^ tumor to be ablated over 30 min, with maximum stomach and blood vessel temperatures of 43.2 and 45.0 °C, respectively.

### Parametric studies of preservation of blood vessels

**Fig. 8:**
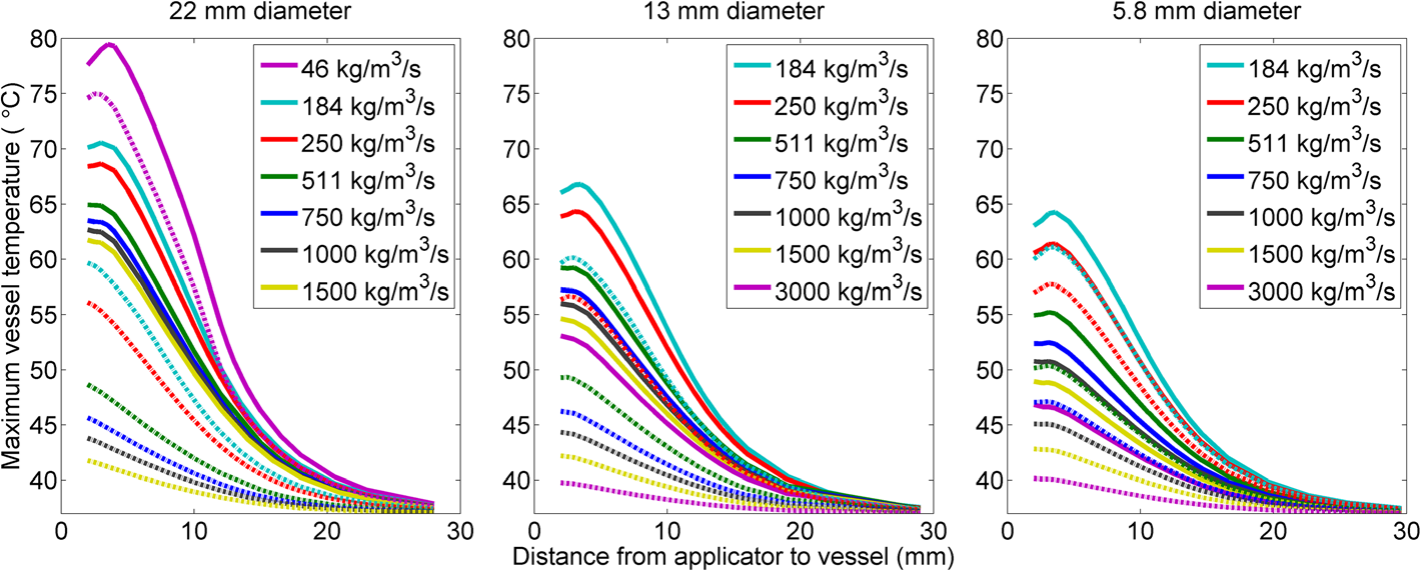
Maximum temperature on the outer (solid) and inner (dashed) surfaces of blood vessels after 10 min of heating, as a function of distance from the applicator to the outer surface of the blood vessel, for a variety of heat transfer coefficients. The temperature of the aorta is given in (**a**), the temperature of large 13.0 mm OD vessels is given in (**b**), and the temperature of small 5.8 mm OD vessels is given in (**c**)

A simulated parametric study was performed to determine the effects of errors in the estimated heat transfer coefficient on calculated heating of blood vessels. Figure 8 shows the maximum temperatures on the inner and outer surfaces of blood vessels of various sizes after 10 min of heating, for a variety of heat transfer coefficients and distances between the transgastric applicator and the blood vessels. For a wide margin of error, that the heat transfer coefficients are at least 25% of the estimated values, acoustic energy can be directed at blood vessels provided that the applicator is positioned at least 15.8 mm from the vena cava, 15.1 mm from the aorta, 14.4 mm from large blood vessels such as the portal vein, or 13.4 mm from small blood vessels such as the superior mesenteric artery, assuming that maximum tissue temperatures are limited to 80 °C. Using the estimated heat transfer coefficients without a margin of error, the applicator should be positioned at least 14.8 mm from the vena cava, 13.9 mm from the aorta, 12.0 mm from large blood vessels, and 9.4 mm from small blood vessels.

Blood vessel temperatures were more sensitive to changes in the heat transfer coefficient when the applicator was closer to the vessels. Changes in heat transfer coefficients also had a larger effect on temperatures in smaller vessels than larger vessels, and this effect was more pronounced on the outer surface on the vessels. As expected, vessel temperatures were higher when the applicator was closer to the vessel, with an exception in some cases with low heat transfer coefficients when the applicator was close enough (<4 mm) for catheter cooling to play a role.

### Parametric study of duodenal heating

**Fig. 9:**
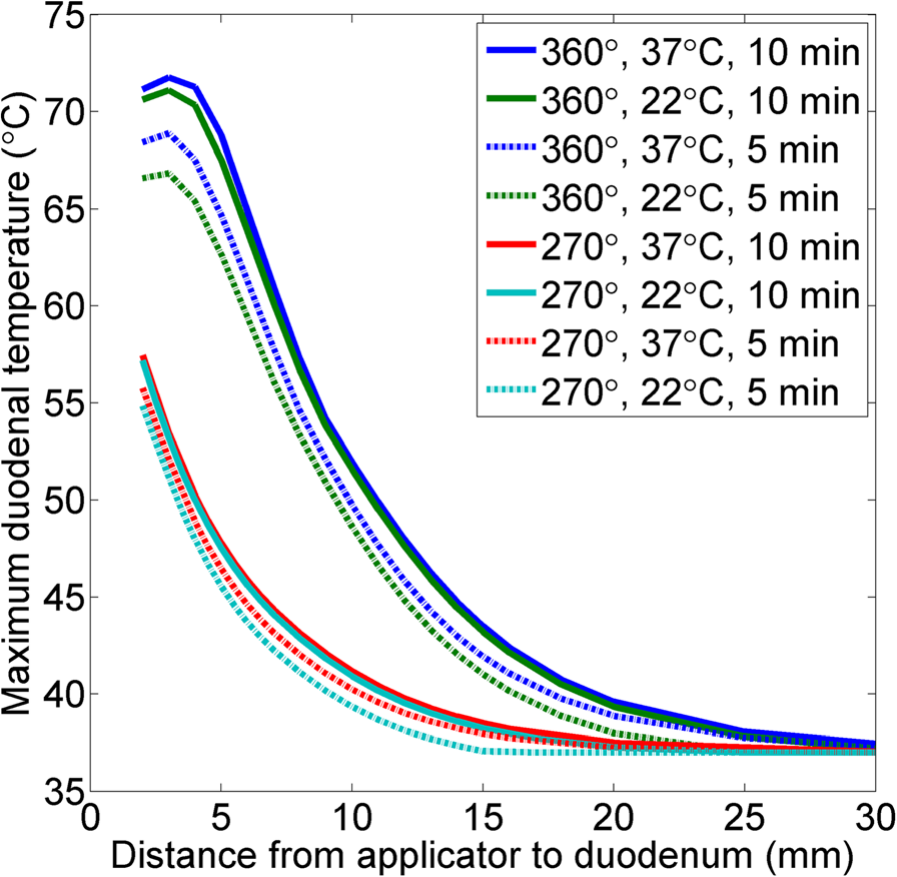
The maximum temperature of the duodenum (°C) when heated by a 360° applicator or a 270° applicator directed away from the duodenum, as a function of distance from the applicator to the duodenum, for 5 and 10 min treatment times and for initial temperatures of the duodenal lumen of 22 °C and 37 °C

A parametric study was performed to determine the treatment parameters necessary for preservation of the duodenum. The maximum temperature on the duodenum for 360° and 270° applicators 2–30 mm from the duodenum is plotted in Fig. 9, with treatment times of 5 and 10 min and initial temperatures of 22 and 37 °C.

The distance from the applicator to the duodenum and the sector size of the applicator had more pronounced effects on the maximum duodenum temperature than initial temperature or treatment time. Use of a 270° applicator aimed directly away from the duodenum decreases the duodenal temperatures up to 21.1 °C when compared to a 360° applicator for a 10 min ablation. A 360° applicator parallel to the duodenum would have to be positioned at least 14 mm from the duodenum to avoid damage (<45 °C) after 10 min of ablation, assuming an initial temperature of 37 °C. A 270° applicator, however, could be placed 7 mm from the duodenum under the same conditions.

Reducing the treatment time from 10 to 5 min had only a moderate effect on duodenal temperatures for 360 °C applicators, with maximum duodenal temperatures decreasing by at most 4.1 °C. For 270° applicators, this reduction in treatment time had even less effect, with a change in maximum duodenal temperatures of only 1.7 °C at most.

Filling of the duodenum with 22 °C cooling water prior to treatment had a negligible effect on the maximum duodenum temperature at the end of 10 min treatments with 360° applicators and treatments of 5 or 10 min with 270° applicators directed away from the duodenum. With short treatment times using 360° applicators, the initial cooling has a small effect. Cooling reduced the maximum duodenum temperature by 2.1 °C or less after 5 min treatments with 360° applicators, and only 1.3 °C or less after 10 min treatments with 360° applicators. For 270° applicators directed away from the duodenum, duodenal cooling reduced the maximum duodenum temperature by only 1.0 and 0.4 °C after 5 and 10 min treatments, respectively.

### Parametric study of absorption and perfusion

**Fig. 10:**
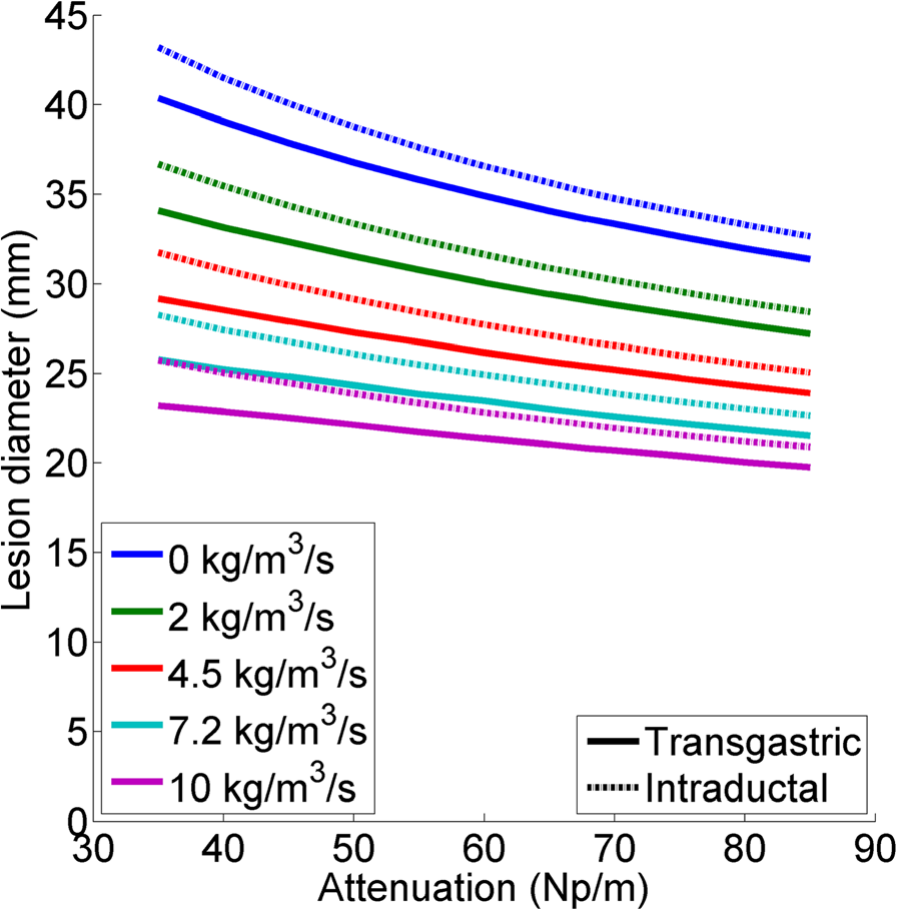
The maximum thermal lesion diameter (T > 52 °C) after 10 min of heating as a function of tissue attenuation, for a variety of blood perfusion rates for both transgastric (solid lines) and intraductal (dashed lines) applicators

The thermal lesion diameter that can be attained after 10 min of heating with transgastric and intraductal applicators is plotted in Fig. 10 for a variety of acoustic attenuation coefficients and blood perfusion rates. Assuming a tumor attenuation of 68 Np/m and perfusion of 4.5 kg/m3/s (Table 1), this study indicated that pancreatic tumors up to 2.5 or 2.7 cm diameter can be ablated within 10 min using the transgastric and intraductal approaches, respectively. Intraductal applicators produced slightly larger (1.1–2.5 mm in diameter) thermal lesions than transgastric applicators, particularly in tissues with lower attenuations. Variations in blood perfusion rates generally had more pronounced effects on thermal lesion diameters than did variations in acoustic attenuation coefficients. Variations in acoustic attenuation had a greater effect on lesion sizes at lower blood perfusion rates than at higher rates.

## Discussion

This study was performed to provide a broad and preliminary investigation of the feasibility of transgastric and intraductal approaches to ultrasound-based thermal therapy of pancreatic tumors. Such an approach would provide localized directionality of heating which RF, microwave, and laser ablation lack, without the acoustic window limitations faced by HIFU. Through patient specific modeling and parametric studies we have demonstrated that this technology would have the ability to treat tumors positioned > 4–5 cm from the gastrointestinal (GI) tract, which ultrasound applicators sonicating from within the tract cannot access. Patient-specific simulations herein have demonstrated the ability of transgastric and intraductal ultrasound to generate therapeutic temperature distributions within the majority of the volumes of pancreatic tumors without damage to sensitive structures. Further, parametric studies identified appropriate treatment parameters for a variety of settings to avoid thermal damage to the duodenum or major blood vessels, and to identify limitations of the technology.

Sensitive tissues can be protected through careful placement of the applicator and through the use of sectored applicators. Guidance of applicator placement may be achieved clinically through MR, CT, ultrasound, or fluoroscopic guidance. Needle-based temperature probes or MR temperature imaging could be applied to confirm correct placement and to ensure that sensitive tissues are not overheated. To prevent overheating in the direction of non-targeted anatomy, transducers with wide sector angles (210° or 270°) were employed in four patient-specific simulations. Wide sector angles allow for heating of large tumor volumes, and only limited heating dominated by conduction occurs in the direction of the sensitive structures. Sectored applicators can be positioned closer to sensitive anatomy than 360° applicators, with the emitted energy directed away from these organs. Filling the duodenum with cold water prior to treatment was not found to be a highly effective means of tissue protection, reducing the duodenal temperature by 2.1 °C or less (Fig. 9). Without active cooling of the duodenum, 360° applicators can be placed 14 mm, and directional applicators can be placed 7 mm, from the duodenal wall without damaging the tissue over the course of a 10 min treatment. The high blood perfusion rates in the duodenum, as well as the relatively high wall thickness considered in order to simulate a worst case scenario, may have contributed to this result.

Applicator placement was determined based on the tumor shape and the location of sensitive structures. In most cases, the applicator was placed in the center of the target volume. If possible, the applicator axis was aligned with the long axis of the tumor, as the ablated zone has the shape of an ellipsoid with the long axis aligned with the applicator. In cases where sensitive anatomy is to one side of the tumor, the applicator could be placed either perpendicular to and outside the surface of the sensitive structure, such that the well-collimated acoustic output does not enter it (Case 8), or off-center within the tumor, similar to the 360° applicator positioned away from sensitive tissues in Case 6 (Fig. 4). An ultrasound applicator can be placed closer to blood vessels, which are self-cooling due to blood flow, than to organs, as can be seen by comparing Figs. 8 and 9. Our finding that a 360° ultrasound applicator should be placed at least 12 mm from large blood vessels like the portal vein is in accordance with a study by Wu et al. [53]. They found that 5 mm is an insufficient safety margin between an RFA site and major peripancreatic vessels, namely the portal vein, in this study in which three deaths were caused by portal vein thrombosis followed by massive gastrointestinal hemorrhage [53].

The selection of either a transgastric or intraductal approach can be decided based on the size and location of the tumor. A transgastric approach can be used in cases with an available route from the stomach or duodenum that does not transverse major blood vessels or sensitive tissues. Depending on the size of the duodenum and the constriction and distance from the pyloric sphincter, attaining the desired insertion angle may be easier from the stomach, where there is greater freedom of motion, than from the duodenum. An intraductal approach is more appropriate for tumors near the head of the pancreas that can be accessed from and are immediately adjacent to or encapsulating the pancreatic duct. The intraductal approach can ablate slightly larger volumes than the transgastric approach (26.8 mm vs. 25.4 mm diameter, assuming 4.5 kg/m^3^/s perfusion and 68 Np/m attenuation). For tumors partially encapsulating the duct, directional sectored applicators may be more appropriate, but they produce much smaller ablated volumes.

The intraductal approach is less invasive than the transgastric one, as it does not require any penetration through the stomach or duodenal wall. Although other transgastric interventions are being investigated with larger instruments than the transgastric ultrasound applicators presented herein [54], less invasive procedures are generally preferable when possible, thus making the intraductal approach more attractive when feasible. However, the full target volume must be accessible (<1.2 cm for ablation from applicator to outer target boundary) from the pancreatic or bile duct. The size of the duct may potentially limit some intraductal treatments to tumors near the head of the pancreas, which is acceptable, as about 70–85% of pancreatic tumors arise in the head [6]. Procedures commonly used for dilatation and placement of stents could be applied during the placement of the ultrasound device.

Hyperthermia for the treatment of pancreatic cancer, as an adjunct to radiation therapy, chemotherapy, immunotherapy, or nanoparticle drug delivery, has potential clinical utility. The feasibility of low temperature heating or hyperthermia delivery is largely dependent on the tumor size and location. Hyperthermia can be applied using the less invasive intraductal approach, following procedures commonly used for pancreatic stenting. Large tumors requiring multiple applicator positions are inappropriate for hyperthermia, as treatment at each position would require 30–60 min. Hyperthermia also has all the limitations associated with the intraductal approach, including less flexibility in selection of applicator positions than the transgastric approach. Hyperthermia may be preferred over ablation for moderately sized tumors with sensitive anatomy on multiple sides of the tumor, as in Case 7, where sensitive tissues could not be readily protected solely by the use of directional transducers.

Larger proportions of tumor volumes were treated with hyperthermia (89.9–94.7%) than with ablation (55.3–83.3%) in this study. This may be due in part to the lower risk of thermal injury to sensitive tissues during hyperthermia placing fewer limitations on treatment, and to the selection of tumors small enough for treatment with a single applicator position. Because hyperthermia uses inherently lower and safer temperatures than ablation, larger proportions of tumor volumes can be treated with less heating of nearby sensitive tissues.

When planning treatments of pancreatic tumors, the attenuation coefficients of the tumor tissue and desmoplastic stroma should be taken into consideration in order to optimize treatment parameters. Parametric studies demonstrate the significant effect tissue attenuation rates have on the size of the ablated region, especially in cases with low blood perfusion rates (Fig. 10). Pancreatic ductal adenocarcinoma, a common form of pancreatic cancer, is known to exhibit extensive and heterogeneous fibrosis [55, 56]. Fibrous tissues generally have higher attenuation coefficients than other soft tissues [52], which could result in preferential heating of the desmoplastic stroma nearest the applicator, and less acoustic propagation through the stroma into the tumor. This could possibly result in reduced heating of both the portion of the tumor further from the applicator, and any sensitive anatomy. A study to obtain measurements of tissue properties for pancreatic tumor samples, as related to their fibrous content and appearance under diagnostic medical imaging, would be extremely useful in informing further development of ultrasound-based therapeutic strategies for pancreatic cancer. Both attenuation coefficients, and the distribution of desmoplastic stroma in and around the targeted tumor, should be taken into account when planning treatments.

The favorable findings of this exploratory study, although preliminary, indicate that further investigation of these approaches for delivering ultrasonic or thermal therapies for the treatment of pancreatic cancer is warranted. The pathway for additional development toward clinical implementation could include the design, fabrication, and experimental evaluations of intraluminal and transgastric devices specific for the pancreas. Specific image guidance approaches, such as MRI with non-invasive temperature monitoring [57–59], or ultrasound or CT imaging with electromagnetic tracking [60–62], could be integrated as a means to precisely position and verify therapy delivery. Incorporation of optimization-based patient specific treatment planning [39, 63, 64] could be applied for a priori or real-time determination of ideal positioning, applicator selection, and applied power trajectories. This could include model-based feedback treatment control, to optimally determine parameters for conformal targeting and ensuring adequate safety zones. Detailed and extensive in vivo studies in large animals could be performed to characterize thermal dosimetry, to closely evaluate heating around ducts and vessels, to define limitations and safety information, to provide feedback to designs and monitoring approaches, and to validate guidance and planning as above. Given successful development within this framework, precise delivery of safe and conformal hyperthermia or thermal ablation of target regions within the pancreas can possibly be delivered in a minimally-invasive fashion, thereby providing a superior alternative to other invasive modalities in regions where extracorporeal HIFU may not be practicable.

## Conclusion

This study has demonstrated the feasibility of ablation of 2.5 cm diameter targets in the pancreas using transgastric and intraductal ultrasound applicators, provided that there is sufficient separation of sonicated regions from sensitive organs and blood vessels. To preserve sensitive structures, 360° applicators should be placed at least 13.9–14.8 mm from major vessels like the aorta or vena cava, 9.4–12.0 mm from other sizable vessels, and 14 mm from the duodenum. Alternatively, sectored transducers can be positioned closer to sensitive anatomy, with the emitted energy directed away from these structures. In cases with tumors near or encapsulating the pancreatic duct, intraductal hyperthermia may provide a therapy option in conjunction with radiation or chemotherapy.

## Abbreviations

3D: Three-dimensional
CT: Computed tomography
GI: Gastrointestinal
HIFU: High intensity focused ultrasound
ID: Inner diameter
MR: Magnetic resonance
OD: Outer diameter
PI: Proportional integral
RFA: Radiofrequency ablation

## References

[1] Siegel R (2014). Cancer statistics, 2014. CA Cancer J Clin.

[2] Morganti AG (2010). A systematic review of resectability and survival after concurrent chemoradiation in primarily unresectable pancreatic cancer. Ann Surg Oncol.

[3] Hidalgo M (2010). Pancreatic cancer. N Engl J Med.

[4] Vincent A (2011). Pancreatic cancer. Lancet.

[5] Keane MG (2014). Systematic review of novel ablative methods in locally advanced pancreatic cancer. World J Gastroenterol.

[6] Brescia FJ (2004). Palliative care in pancreatic cancer. Cancer Control.

[7] Cantore M (2012). Combined modality treatment for patients with locally advanced pancreatic adenocarcinoma. Br J Surg.

[8] Khokhlova TD, Hwang JH (2011). HIFU for palliative treatment of pancreatic cancer. J Gastrointest Oncol.

[9] D’Onofrio M (2010). Radiofrequency ablation of locally advanced pancreatic adenocarcinoma: an overview. World J Gastroenterol.

[10] Arcidiacono PG (2012). Feasibility and safety of EUS-guided cryothermal ablation in patients with locally advanced pancreatic cancer. Gastrointest Endosc.

[11] Pai M (2013). Endoscopic Ultrasound Guided Radiofrequency Ablation (EUS-RFA) for Pancreatic Ductal Adenocarcinoma. Gut.

[12] Li T (2015). Endoscopic high-intensity focused US: technical aspects and studies in an in vivo porcine model (with video). Gastrointest Endosc.

[13] Adams MS (2016). Thermal therapy of pancreatic tumours using endoluminal ultrasound: Parametric and patient-specific modelling. Int J Hyperth.

[14] Salgaonkar VA, Diederich CJ (2015). Catheter-based ultrasound technology for image-guided thermal therapy: Current technology and applications. Int J Hyperth.

[15] Nau WH (2005). MRI-guided interstitial ultrasound thermal therapy of the prostate: A feasibility study in the canine model. Med Phys.

[16] Prakash P (2012). Multiple applicator hepatic ablation with interstitial ultrasound devices: Theoretical and experimental investigation. Med Phys.

[17] Scott JS, Prakash P, Salgaonkar V, Jones PD, Cam RN, Han M, Rieke V, Burdette EC, Diederich CJ. Interstitial ultrasound ablation of tumors within or adjacent to bone: Contributions of preferential heating at the bone surface. Proc. SPIE 8584, Energy-based Treatment of Tissue and Assessment VII, 85840Z. 2013. doi:10.1117/12.2002632.

[18] Scott SJ (2014). Interstitial ultrasound ablation of vertebral and paraspinal tumors: Parametric and patient specific simulations. Int J Hyperth.

[19] Kangasniemi M (2002). Multiplanar MR temperature-sensitive imaging of cerebral thermal treatment using interstitial ultrasound applicators in a canine model. J Magn Reson Imaging.

[20] Pauly KB (2006). Magnetic resonance-guided high-intensity ultrasound ablation of the prostate. Top Magn Reson Imaging.

[21] Diederich CJ, Moros E (2012). Endocavity and catheter-based ultrasound devices. Physics of Thermal Therapy: Fundamentals and Clinical Applications.

[22] Deardorff DL, Diederich CJ, Nau WH (2000). Control of interstitial thermal coagulation: Comparative evaluation of microwave and ultrasound applicators. Med Phys.

[23] Deardorff DL, Diederich CJ (2000). Axial control of thermal coagulation using a multi-element interstitial ultrasound applicator with internal cooling. IEEE Trans Ultrason Ferroelectr Freq Control.

[24] Nau WH, Diederich CJ, Stauffer PR (2000). Directional power deposition from direct-coupled and catheter-cooled interstitial ultrasound applicators. Int J Hyperth.

[25] Steel AW (2011). Endoscopically applied radiofrequency ablation appears to be safe in the treatment of malignant biliary obstruction. Gastrointest Endosc.

[26] Wadsworth CA, Westaby D, Khan SA (2013). Endoscopic radiofrequency ablation for cholangiocarcinoma. Curr Opin Gastroenterol.

[27] Figueroa-Barojas P. et al., Safety and efficacy of radiofrequency ablation in the management of unresectable bile duct and pancreatic cancer: a novel palliation technique. J Oncol. 2013;2013:1-5. 10.1155/2013/910897 PMC3649248 23690775

[28] Prat F (2002). Endoscopic treatment of cholangiocarcinoma and carcinoma of the duodenal papilla by intraductal high-intensity US: Results of a pilot study. Gastrointest Endosc.

[29] Yoon WJ, Brugge WR (2012). Endoscopic ultrasonography-guided tumor ablation. Gastrointest Endosc Clin N Am.

[30] Pai M (2015). Endoscopic ultrasound guided radiofrequency ablation, for pancreatic cystic neoplasms and neuroendocrine tumors. World J Gastrointest Surg.

[31] Carrara S (2013). Tumors and new endoscopic ultrasound-guided therapies. World J Gastrointest Endosc.

[32] Prakash P, Salgaonkar VA, Diederich CJ (2013). Modelling of endoluminal and interstitial ultrasound hyperthermia and thermal ablation: Applications for device design, feedback control and treatment planning. Int J Hyperth.

[33] Diederich CJ, Hynynen K (1989). Induction of hyperthermia using an intracavitary multielement ultrasonic applicator. IEEE Trans Biomed Eng.

[34] Tschoep-Lechner KE (2013). Gemcitabine and cisplatin combined with regional hyperthermia as second-line treatment in patients with gemcitabine-refractory advanced pancreatic cancer. Int J Hyperthermia.

[35] Ishikawa T (2012). Phase II trial of combined regional hyperthermia and gemcitabine for locally advanced or metastatic pancreatic cancer. Int J Hyperth.

[36] Brugge WR (2009). EUS-guided tumor ablation with heat, cold, microwave, or radiofrequency: will there be a winner?. Gastrointest Endosc.

[37] Nau WH, Diederich CJ, Burdette EC (2001). Evaluation of multielement catheter-cooled interstitial ultrasound applicators for high-temperature thermal therapy. Med Phys.

[38] Diederich CJ (1996). Ultrasound applicators with integrated catheter-cooling for interstitial hyperthermia: Theory and preliminary experiments. Int J Hyperth.

[39] Chen X (2010). Optimisation-based thermal treatment planning for catheter-based ultrasound hyperthermia. Int J Hyperth.

[40] Scott SJ (2013). Approaches for modeling interstitial ultrasound ablation of tumors within or adjacent to bone: Theoretical and experimental evaluations. Int J Hyperth.

[41] Ross AB (2004). Highly directional transurethral ultrasound applicators with rotational control for MRI-guided prostatic thermal therapy. Phys Med Biol.

[42] Hadidi A (1983). Pancreatic duct diameter: Sonographic measurement in normal subjects. J Clin Ultrasound.

[43] Pfau PR (2013). Pancreatic and biliary stents. Gastrointest Endosc.

[44] Tantau M (2008). Intraductal ultrasonography for the assessment of preoperative biliary and pancreatic strictures. J Gastrointest Liver Dis.

[45] Pennes HH (1948). Analysis of tissue and arterial blood temperatures in the resting human forearm. J Appl Physiol.

[46] Kinsey AM (2008). Transurethral ultrasound applicators with dynamic multi-sector control for prostate thermal therapy: In vivo evaluation under MR guidance. Med Phys.

[47] Dewhirst MW (2003). Basic principles of thermal dosimetry and thermal thresholds for tissue damage from hyperthermia. Int J Hyperthermia.

[48] Haemmerich D (2003). Hepatic bipolar radiofrequency ablation creates coagulation zones close to blood vessels: A finite element study. Med Biol Eng Comput.

[49] Cronin CG (2010). Normal small bowel wall characteristics on MR enterography. Eur J Radiol.

[50] Fleischer AC, Muhletaler CA, James A (1981). Sonographic assessment of the bowel wall. Am J Roentgenol.

[51] Vaupel P, Kallinowski F, Okunieff P (1989). Blood flow, oxygen and nutrient supply, and metabolic microenvironment of human tumors: A review. Cancer Res.

[52] Duck F (1990). Physical Properties of Tissue: A Comprehensive Reference Book.

[53] Wu Y (2006). High operative risk of cool‐tip radiofrequency ablation for unresectable pancreatic head cancer. J Surg Oncol.

[54] Shaikh SN, Thompson CC (2010). Natural orifice translumenal surgery: Flexible platform. World J Gastrointest Surg.

[55] Whatcott C (2013). Tumor-stromal interactions in pancreatic cancer. Crit Rev Oncog.

[56] Verbeke C (2016). Morphological heterogeneity in ductal adenocarcinoma of the pancreas - Does it matter?. Pancreatology.

[57] Bing C (2016). Drift correction for accurate PRF-shift MR thermometry during mild hyperthermia treatments with MR-HIFU. Int J Hyperthermia.

[58] de Senneville BD (2007). MR thermometry for monitoring tumor ablation. Eur Radiol.

[59] Rieke V, Butts Pauly K (2008). MR thermometry. J Magn Reson Imaging.

[60] Ebbini ES, ter Haar G (2015). Ultrasound-guided therapeutic focused ultrasound: current status and future directions. Int J Hyperthermia.

[61] Sanchez Y et al. Navigational Guidance and Ablation Planning Tools for Interventional Radiology. Curr Probl Diagn Radiol. 2016;1–9. 10.1067/j.cpradiol.2016.11.002 28041657

[62] Rajagopal M, Venkatesan AM (2016). Image fusion and navigation platforms for percutaneous image-guided interventions. Abdom Radiol (NY).

[63] Prakash P, Diederich CJ (2012). Considerations for theoretical modelling of thermal ablation with catheter-based ultrasonic sources: Implications for treatment planning, monitoring and control. Int J Hyperth.

[64] Paulides MM (2013). Simulation techniques in hyperthermia treatment planning. Int J Hyperth.

[65] Bamber J, Hill C (1981). Acoustic properties of normal and cancerous human liver—I. Dependence on pathological condition. Ultrasound Med Biol.

[66] Kandel S (2009). Whole-organ perfusion of the pancreas using dynamic volume CT in patients with primary pancreas carcinoma: acquisition technique, post-processing and initial results. Eur Radiol.

[67] Delrue L (2012). Tissue perfusion in pathologies of the pancreas: assessment using 128-slice computed tomography. Abdom Imaging.

[68] Gerweck LE (1985). Hyperthermia in cancer therapy: The biological basis and unresolved questions. Cancer Res.

[69] Mcintosh RL, Anderson V (2010). A comprehensive tissue properties database provided for the thermal assessment of a human at rest. Biophys Rev Lett.

[70] Giering K, Lamprecht I, Minet O. Specific heat capacities of human and animal tissues. Proc. SPIE 2624, Laser-Tissue Interaction and Tissue Optics, 188 (January 10, 1996). doi:10.1117/12.229547; http://dx.doi.org/10.1117/12.229547.

[71] Phillips M (2012). Irreversible electroporation on the small intestine. Br J Cancer.

[72] Mott HA (1877). Chemists’ manual: A practical treatise on chemistry, qualitative and quantitative analysis, stoichiometry, blowpipe analysis, mineralogy, assaying, toxicology, etc., etc., etc.

[73] Werner J, Buse M (1988). Temperature profiles with respect to inhomogeneity and geometry of the human body. J Appl Physiol.

[74] Williams LR, Leggett RW (1989). Reference values for resting blood flow to organs of man. Clin Phys Physiol Meas.

[75] Wootton JH (2011). Implant strategies for endocervical and interstitial ultrasound hyperthermia adjunct to HDR brachytherapy for the treatment of cervical cancer. Phys Med Biol.

[76] Poutanen T (2003). Normal aortic dimensions and flow in 168 children and young adults. Clin Physiol Funct Imaging.

[77] Gabe IT (1969). Measurement of instantaneous blood flow velocity and pressure in conscious man with a catheter-tip velocity probe. Circulation.

[78] Wexler L (1968). Velocity of blood flow in normal human venae cavae. Circ Res.

[79] Arienti V (1996). Doppler ultrasonographic evaluation of splanchnic blood flow in coeliac disease. Gut.

[80] Zironi G (1992). Value of measurement of mean portal flow velocity by Doppler flowmetry in the diagnosis of portal hypertension. J Hepatol.

[81] Perišić MD, Ćulafić DM, Kerkez M (2005). Specificity of splenic blood flow in liver cirrhosis. Rom J Intern Med.

[82] Sato S (1987). Splenic artery and superior mesenteric artery blood flow: nonsurgical Doppler US measurement in healthy subjects and patients with chronic liver disease. Radiology.

